# Changes in incidence and prognosis of malignancies in children with HIV

**DOI:** 10.1186/1750-9378-7-S1-P2

**Published:** 2012-04-19

**Authors:** Daniela C Stefan, David K Stones, Rob Newton

**Affiliations:** 1 Department of Paediatrics and Child Health, Tygerberg Children’s Hospital ,Stellenbosch University, Cape Town, South Africa; 2 Department of Paediatrics and Child Health, Universitas, Bloemfontein, South Africa; 3 Department of Epidemiology, York University, York, UK

## Aim

The aim of this study was to analyze the differences in patient demographics as well as in the relative incidence and outcome of childhood cancers, associated with the HIV infection.

## Material and methods

A retrospective comparative study of two series of children with malignant disease, one with HIV one without, was carried out. The former series consisted of 99 African children with cancer and HIV, consecutively admitted at Tygerberg Children’s Hospital, Cape Town and Universitas Hospital, Bloemfontein, from 1995 to 2010. The latter series was formed of 570 African children with malignant diseases, not infected with HIV, consecutively admitted at the 2 hospitals, from January 2002 to December 2010. Variables studied were age, sex ratio, distribution of various malignancies, length of follow-up, treatment abandonment and mortality.

## Results

The HIV positive children tended to be younger at diagnosis. The male/female ratio was slightly over 2 to 1 in the HIV positive group, while in the control group the sex ratio approached 1:1. Kaposi sarcoma was seen exclusively in the HIV positive series.

The death rate was 50.5% in the HIV positive children (versus 40.8% in HIV negative) but the difference is not significant.

When subgroups with matched cancers were compared, children infected with HIV had a significantly higher risk to die of drug-induced toxicity (relative risk 29.2, 95% confidence interval 3.7-225.8); only 26% of the HIV-positive children survived, compared with 51.2% in those not HIV infected (p=0.02).

## Conclusions

The infection with HIV increases the risk for Kaposi sarcoma, for death due to cytostatic toxicity as well as the overall risk of death in children with cancer.

